# Risk of falls and fear of falling in older adults residing in public housing in Ontario, Canada: findings from a multisite observational study

**DOI:** 10.1186/s12877-019-1399-1

**Published:** 2020-01-09

**Authors:** Melissa Pirrie, Guneet Saini, Ricardo Angeles, Francine Marzanek, Jenna Parascandalo, Gina Agarwal

**Affiliations:** 1 Department of Family Medicine, McMaster University, David Braley Health Sciences Centre, 100 Main Street West, 5th Floor, Hamilton, ON L8P 1H6 Canada; 2 Department of Health Research Methods, Evidence, and Impact, McMaster University, 1280 Main Street West, Hamilton, ON L8S 4L8 Canada

**Keywords:** Older adults, Public housing, Fall risk, Fear of falling, Low income, Social determinants of health

## Abstract

**Background:**

Falls in older adults is a widely researched topic. However, older adults residing in public housing are a vulnerable population that may have unique risk factors for falls. This study aims to describe the prevalence and risk factors for falls, fear of falling, and seeking medical attending for falls in this population.

**Methods:**

Sociodemographic and health-related data was collected as part of a community-based health assessment program with older adults in public housing. Three pre-screening questions identified individuals at potential risk for falls; individuals who screened positive performed the objective Timed Up and Go (TUG) test. Logistic regression was used to evaluate risk factors for four outcome variables: falls in the past year, seeking medical attention for falls, fear of falling, and objectively measured fall risk via TUG test.

**Results:**

A total of 595 participants were evaluated, of which the majority were female (81.3%), white (86.7%), did not have a high school diploma (50.0%), and reported problems in mobility (56.2%). The prevalence of falls in the past year was 34.5%, seeking medical attention for falls was 20.2% and fear of falling was 38.8%. The TUG test was completed by 257 participants. Notably, males had significantly reduced odds of seeking medical attention for a fall (OR = 0.50, 95%CI 0.25–0.98) and having a fear of falling (OR = 0.42, 95%CI 0.24–0.76); daily fruit and vegetable consumption was associated with decreased odds of having a fall in the past year (OR = 0.55, 95%CI 0.37–0.83), and alcohol consumption was associated with increased odds of fear of falling (OR = 1.72, 95%CI 1.03–2.88).

**Conclusion:**

Older adults residing in public housing have unique risk factors associated with social determinants of health, such as low fruit and vegetable consumption, which may increase their risk for falls. The findings of this study can be used to inform falls interventions for this population and identify areas for further research.

## Background

According to the 2008–2009 Canadian Community Health Survey, approximately 1 in 5 Canadians over the age of 65 experienced a fall in the previous year [1]. In those who sustained an injury, approximately 45.1% received medical attention and 26.6% required hospitalization. Some of the injuries included bone fractures (8.4%), head injuries (3.0%) and sprains or strains (9.9%) [1]. Compared to the length of stay for seniors admitted for all causes, fall-related hospitalization is, on average, nine days longer [2]. Additionally, older adults hospitalized for a fall are more likely to be moved into long-term care than those hospitalized for other reasons [3]. This results in a loss of independence for the individual and increased costs for the healthcare system due to fall-related injuries.

The prevalence of falls and ‘fear of falling’ have been extensively studied in the general population of community-dwelling older adults. Previous studies have found factors such as falls in the previous year, age, gender, ethnicity, educational level, body mass index, household income, chronic diseases, depression, pain and visual impairment to be associated with fear of falling [4, 5]. In a systematic review, Deandrea et al. [6] examined the association of over 30 factors with risk of falling as identified within 74 prospective cohort studies. Sociodemographic variables such as age, sex, history of falls, physical activity, physical disability, visual impairment, instrumental disability, education, and walking aid use were associated with falls [6]. Moreover, medical and psychological factors including depression, comorbidities, antihypertensive medication, self-perceived poor health, fear of falling and Parkinson disease were associated with an increased risk of falling [6].

Older adults living in public housing are a vulnerable population with low socioeconomic status, low education, and low health literacy [7]. Low-income older adults report poorer physical and mental health compared to the general population of community-dwelling older adults [7]. Based on the available studies [4–6], it is expected there will be a higher prevalence of falls in this population, but this remains a gap in the literature. Also, the risk factors associated with falling and fear of falling in this population may deviate from those observed in the general population, but this has not been evaluated. Finally, low-income older adults may be more likely to seek medical attention based on their general patterns of health care utilization [3, 8, 9]. The association between low socioeconomic status (SES) and a high number of emergency department (ED) visits and subsequent hospitalization has been well documented among community-dwelling elderly [8, 9]. It has also been shown that older adults with a lower SES have a longer length of hospitalization than those with a high SES and having poor health prior to hospitalization predicts a worse trajectory post-fall [3, 9]. Therefore, it is important to determine the prevalence of and risk factors for falls, fear of falling, and seeking medical attention for falls in this vulnerable population.

Using baseline data from an existing cardiovascular health assessment program across Ontario, this cross-sectional study aimed to better understand the prevalence of falling, receiving medical attention for falls, fear of falling and objectively measured fall risk among older adults residing in public housing. Additionally, the risk factors associated with each of these outcomes was determined, an area of research that is currently unexplored. The findings from this study could be used to inform and evaluate safety and falls prevention initiatives for older adults that are offered through public health programming.

## Methods

### Study setting and participants

Using a cross-sectional design, this study used baseline data collected through assessments as part of the randomized control trial for the Community Paramedicine at Clinic (CP@clinic) program [10]. CP@clinic is a weekly health promotion and health prevention drop-in program designed to target low-income seniors in public housing buildings, in order to assess modifiable risk factors for health conditions, educate participants and then link them to resources in their community.

The participants in this study consisted of older adults aged 55 and older residing in public housing buildings within five communities in Ontario, Canada (Hamilton, Guelph, Simcoe County, Sudbury, and York) who underwent health assessments by community paramedics from 2013 to 2015. A total of 595 residents attended the CP@clinic program. The study population represents community-dwelling adults of low socioeconomic status in public housing. For most Ontario public housing providers, 55 is the minimum age for residing in buildings designated for seniors.

### Measures

All CP@clinic participants were asked three screening questions recommended for identifying fall risk in older adults by primary care: “Have you fallen in the last year?”, “Have you sought medical attention for a fall?” and “Do you have a fear of falling?” [11, 12]. Participants who answered yes to at least one question were objectively assessed for fall risk using the Timed Up and Go (TUG) test, a validated screening measure for identifying older adults at risk of falling [13]. This test consists of an individual being initially seated in a chair, standing up, walking to a marked line 3 m away, turning around, walking back to the chair, and sitting down. If the participant failed to complete this test within 14 s, they were considered to be at risk of falling. TUG test scores (in seconds) were recoded as either at risk (over 14 s) or not at risk (14 s or less) [11, 12].

Health-related indicators collected by the paramedics included self-reported heart disease history, stroke history, hypertension, high cholesterol, diabetes, number of medications, fruit and vegetable intake (≥1 serving per day), salt intake (never, rarely, sometimes, often, always), physical activity (≥30 min of brisk walking daily), current smoking status, alcohol consumption (any versus none), body mass index (calculated from objectively measured weight and self-reported height), general self-reported health status (SRHS; poor, fair, good, very good/excellent), health-related quality of life (HRQoL) measures, and whether the participant had a family doctor. HRQoL measures were the five domains from the EQ-5D-3 L instrument: mobility, self-care, usual activities, pain/discomfort, and anxiety/depression [14]. All domains of HRQoL were categorized as “any problems” versus “none.” For a detailed description of the assessments performed by the paramedics, see study protocol for the randomized controlled trial [10].

### Analysis

Descriptive statistics were used to analyze the sociodemographic characteristics, health behaviours, and fall-related outcomes of participants. Binary logistic regression models were created to analyze the association between the four outcome variables (‘had fall in the past year’, ‘ever sought medical attention for a fall’, ‘fear of falling’ and objectively measured ‘fall risk’ via TUG Test), and the independent variables, consisting of sociodemographic factors, including age, gender, ethnicity, level of education, and living alone, and the health-related indicators described above. For the model with ‘fear of falling’ as the outcome variable, ‘had fall in the past year’ was also included as an independent variable due to its association with ‘fear of falling’ in previous literature. Some variables, such as educational level, ethnicity, SRHS, and the HRQoL measures, were collapsed into fewer categories to account for small sample sizes observed with some response options and to minimize the number of variables in the models. Participants with missing responses for any of the independent variables were excluded from the analyses. All analyses were performed using IBM SPSS Statistics 17.0.

## Results

A total of 595 participants (mean age = 73.23 ± 9.18 years) were included in this study. The majority of the full participant group was female (81.3%), white (86.7%), did not have a high school diploma (50.0%), reported problems in mobility (56.2%), experienced some pain or discomfort (69.2%), had hypertension (63.5%), and was obese (42.4%). Based on the three screening questions, 326 participants were identified as being potentially at risk for falls. Of the 257 participants who completed the TUG test, 62 (26.8%) were classified as “at-risk of falling” based on their TUG score. Reasons for not completing the TUG test varied, including concern for participant safety (e.g. relies on wheelchair for ambulation or has limited vision), temporary ailments (e.g. broken leg or recovering from surgery), or participant refusal (e.g. already had a walker and felt further evaluation was unnecessary). Among participants who completed the TUG test, 58.4% had fallen in the past year, 36.2% had ever sought medical attention for a fall and 65.8% had a fear of falling, compared to 34.5, 20.2 and 38.8%, respectively, among all participants. Please see Table 1 for participant characteristics.

**Table 1:** Demographic characteristics and health status indicators of all participants, participants who completed the TUG test, and participants at risk of falling

Variable	All Participants (*n* = 595)	Completed TUG Test (*n* = 257)	At Risk of Falling (*n* = 62)
	n (%)^a^	n (%)^b^	n (%)^c^
Gender			
Male	111 (18.7)	47 (18.3)	16 (25.8)
Female	484 (81.3)	210 (81.7)	46 (74.2)
Age			
55–64	127 (21.3)	52 (20.2)	13 (21.0)
65–74	215 (36.1)	90 (35.0)	14 (22.6)
75–84	195 (32.8)	86 (33.5)	19 (30.6)
85+	58 (9.7)	29 (11.3)	16 (25.8)
Ethnicity			
White	516 (86.7)	237 (92.2)	57 (91.9)
Other	79 (13.3)	20 (7.8)	5 (8.1)
Education			
Some high school	289 (50.0)	134 (52.3)	37 (59.7)
High school diploma	116 (20.1)	48 (18.8)	14 (22.6)
Some or completed post-secondary	173 (29.9)	74 (28.9)	11 (17.7)
Lives Alone	532 (90.8)	232 (90.6)	56 (90.3)
Fallen in the Past Year	205 (34.5)	150 (58.4)	38 (61.3)
Ever Sought Medical Attention for a Fall	120 (20.2)	93 (36.2)	30 (48.4)
Has a Fear of Falling	231 (38.8)	169 (65.8)	50 (80.6)
Mobility Issues	323 (56.2)	159 (63.1)	55 (90.2)
Self-Care Issues	118 (20.6)	61 (24.3)	29 (47.5)
Issues Performing Usual Activities	225 (39.2)	116 (46.0)	44 (72.1)
Pain/Discomfort	398 (69.2)	185 (73.4)	54 (88.5)
Anxiety/Depression	273 (47.6)	137 (54.4)	42 (68.9)
Number of Medications			
None	99 (16.6)	25 (9.7)	5 (8.1)
1–4	159 (26.7)	64 (24.9)	5 (8.1)
5–9	212 (35.6)	105 (40.9)	24 (38.7)
10+	125 (21.0)	63 (24.5)	28 (45.2)
Perceived Health State			
Poor	57 (9.8)	31 (12.1)	14 (22.6)
Fair	162 (27.9)	82 (31.9)	23 (37.1)
Good	222 (38.3)	97 (37.7)	19 (30.6)
Very Good/Excellent	139 (24.0)	47 (18.3)	6 (9.7)
Diabetes	174 (29.8)	85 (33.1)	29 (46.8)
Heart Disease	120 (20.6)	55 (21.4)	20 (32.3)
Hypertension	366 (63.5)	169 (66.0)	46 (74.2)
High Cholesterol	282 (48.5)	138 (53.7)	34 (54.8)
Stroke	53 (9.1)	25 (9.7)	9 (14.5)
Physically Active (30 min per day)	301 (50.6)	130 (50.6)	29 (46.8)
Fruits and Vegetables Everyday	335 (56.3)	119 (46.3)	31 (50.0)
Salt Intake			
Never	201 (34.7)	95 (37.1)	28 (45.2)
Rarely	134 (23.1)	50 (19.5)	10 (16.1)
Sometimes	105 (18.1)	45 (17.6)	14 (22.6)
Often	55 (9.5)	26 (10.2)	2 (3.2)
Always	85(14.7)	40 (15.6)	8 (12.9)
Current Smoker	150 (25.8)	70 (27.2)	10 (16.1)
Consumes Any Alcohol	114 (19.6)	61 (23.8)	12 (19.4)
Has a Family Doctor	527 (88.6)	234 (91.1)	59 (95.2)
Body Mass Index			
Normal	141 (24.2)	62 (24.1)	14 (22.6)
Underweight	18 (3.1)	10 (3.9)	1 (1.6)
Overweight	177 (30.4)	72 (28.0)	15 (24.2)
Obese	247 (42.4)	113 (44.0)	32 (51.6)

Note: *TUG* Timed Up and Go Test, an objective assessment of fall risk; ^a^ missing data was less than 3.6% for each variable; ^b^ missing data was less than 2.4% for each variable; ^c^ missing data was less than 1.7% for each variable

As described in Table 2, the binary logistic regression models revealed significant factors associated with each of the outcomes. The odds of having a fall in the previous year was significantly higher for individuals with mobility issues (OR = 1.75, 95% CI 1.11–2.75) and significantly lower in individuals who consumed at least one serving of fruits and vegetables everyday (OR = 0.55, 95% CI 0.37–0.83) or reported very good/excellent SRHS compared to those with poor SRHS (OR = 0.44, 95% CI 0.21–0.97). The odds of seeking medical attention for a fall was significantly higher for individuals with mobility issues (OR = 2.78, 95% CI 1.58–4.87) and significantly lower in individuals who were male (OR = 0.50, 95% CI 0.25–0.98) or who reported fair (OR = 0.38, 95% CI 0.18–0.81), good (OR = 0.43, 95% CI 0.20–0.89) or very good/excellent (OR = 0.34, 95% CI 0.14–0.80) SRHS. The odds of having a fear of falling was significantly higher in individuals with mobility issues (OR = 1.66, 95% CI 1.05–2.62), those 85 years of age or older (OR = 2.76, 95% CI 1.19–6.40), had any alcohol consumption (OR = 1.72, 95% CI 1.03–2.88), or had fallen in the past year (OR = 2.33, 95% CI 1.55–3.50). Additionally, the odds of having a fear of falling was significantly lower in those who were male (OR = 0.42, 95% CI 0.24–0.76) or reported having a SRHS of good (OR = 0.48, 95% CI 0.24–0.99) or very good/excellent (OR = 0.42, 95% CI 0.19–0.93). Finally, mobility issues (OR = 3.68, 95% CI 1.12–12.60) and the presence of pain and discomfort (OR = 4.56, 95% CI 1.31–15.92) were significantly associated with higher fall risk among participants identified by the TUG test.

**Table 2:** Odds ratios from binary logistic regression models for each fall-related outcome in older adults residing in public housing

Variable	Ever sought medical attention for a fall	Had fall in the past year	Has fear of falling	At risk of falls^a^
	Yes (*n* = 116) vs No (*n* = 441)	Yes (*n* = 196) vs No (*n* = 361)	Yes (*n* = 223) vs No (*n* = 334)	Yes (*n* = 61) vs No (*n* = 170)
	OR (95% CI)	OR (95% CI)	OR (95% CI)	OR (95% CI)
Gender				
Female	REF	REF	REF	REF
Male	**0.50 (0.25–0.98)**	0.99 (0.58–1.69)	**0.42 (0.24–0.76)**	1.80 (0.45–4.45)
Age				
55–64	REF	REF	REF	REF
65–74	0.81 (0.43–1.53)	0.76 (0.45–1.30)	0.94 (0.54–1.63)	0.45 (0.13–1.55)
75–84	0.83 (0.42–1.63)	0.84 (0.47–1.49)	1.18 (0.65–2.15)	1.58 (0.44–5.70)
85+	0.89 (0.35–2.24)	0.84 (0.37–1.87)	**2.76 (1.19–6.40)**	4.07 (0.77–21.50)
Ethnicity				
White	REF	REF	REF	REF
Other	0.53 (0.22–1.29)	0.54 (0.27–1.07)	0.87 (0.44–1.71)	3.26 (0.60–17.61)
Education level				
Some high school	REF	REF	REF	REF
High school diploma	1.14 (0.63–2.07)	0.73 (0.44–1.23)	0.95 (0.57–1.61)	0.67 (0.22–2.04)
Any post-secondary	1.23 (0.72–2.08)	0.75 (0.48–1.19)	0.91 (0.57–1.45)	0.46 (0.15–1.37)
Does not live alone	1.31 (0.53–3.24)	1.90 (0.90–4.02)	0.77 (0.37–1.59)	1.04 (0.21–5.06)
Number of medications				
0	REF	REF	REF	REF
1–4	1.18 (0.55–2.53)	0.69 (0.37–1.27)	0.67 (0.35–1.29)	0.23 (0.03–1.70)
5–9	0.93 (0.46–1.91)	0.57 (0.32–1.02)	1.51 (0.82–2.78)	1.05 (0.20–5.36)
10+	1.08 (0.48–2.41)	1.00 (0.52–1.95)	1.70 (0.84–3.41)	1.62 (0.30–8.80)
Physically active daily	1.06 (0.67–1.67)	1.17 (0.79–1.73)	0.89 (0.60–1.33)	2.09 (0.88–4.96)
Fruits and vegetables daily	0.66 (0.41–1.07)	**0.55 (0.37–0.83)**	0.80 (0.52–1.22)	0.85 (0.34–2.10)
Salt intake				
Never	REF	REF	REF	REF
Rarely	0.98 (0.54–1.77)	1.07 (0.65–1.76)	0.79 (0.47–1.32)	0.39 (0.12–1.22)
Sometimes	1.23 (0.66–2.30)	0.87 (0.50–1.52)	0.80 (0.45–1.43)	2.12 (0.66–6.81)
Often	0.96 (0.40–2.30)	1.37 (0.68–2.79)	1.05 (0.50–2.20)	0.29 (0.05–1.86)
Always	0.85 (0.42–1.76)	0.75 (0.41–1.38)	1.16 (0.63–2.13)	0.47 (0.13–1.70)
Body Mass Index				
Normal	REF	REF	REF	REF
Underweight	0.93 (0.25–3.45)	0.93 (0.30–2.95)	2.90 (0.85–9.91)	1.28 (0.08–20.76)
Overweight	0.70 (0.37–1.33)	0.95 (0.56–1.62)	1.15 (0.66–2.01)	0.94 (0.27–3.26)
Obese	0.63 (0.34–1.18)	0.64 (0.38–1.09)	1.31 (0.75–2.28)	2.38 (0.74–7.67)
Self-reported health				
Poor	REF	REF	REF	REF
Fair	**0.38 (0.18–0.81)**	0.72 (0.36–1.44)	0.65 (0.32–1.34)	0.54 (0.15–2.00)
Good	**0.43 (0.20–0.89)**	0.67 (0.34–1.32)	**0.48 (0.24–0.99)**	0.50 (0.13–2.03)
Very good/excellent	**0.34 (0.14–0.80)**	**0.44 (0.21–0.97)**	**0.42 (0.19–0.93)**	0.43 (0.09–2.13)
Has heart disease	1.51 (0.88–2.58)	1.16 (0.72–1.86)	0.61 (0.37–1.01)	1.45 (0.54–3.89)
Has hypertension	1.06 (0.64–1.76)	0.90 (0.59–1.39)	0.89 (0.57–1.38)	1.38 (0.53–3.56)
Has high cholesterol	1.21 (0.75–1.95)	1.30 (0.86–1.96)	1.43 (0.94–2.17)	0.44 (0.17–1.13)
Has diabetes	1.24 (0.72–2.13)	1.34 (0.84–2.13)	0.75 (0.46–1.23)	1.30 (0.50–3.35)
Has stroke history	0.64 (0.28–1.45)	0.88 (0.45–1.70)	1.25 (0.63–2.48)	1.78 (0.44–7.26)
Has mobility issues	**2.78 (1.58–4.87)**	**1.75 (1.11–2.75)**	**1.66 (1.05–2.62)**	**3.68 (1.12–12.60)**
Has self-care issues	1.50 (0.83–2.73)	1.39 (0.82–2.37)	1.36 (0.78–2.35)	1.81 (0.63–5.19)
Has usual care issues	0.87 (0.50–1.52)	0.85 (0.53–1.38)	1.02 (0.62–1.65)	1.39 (0.51–3.84)
Has pain/discomfort issues	0.89 (0.52–1.53)	1.14 (0.73–1.80)	1.23 (0.77–1.95)	**4.56 (1.31–15.92)**
Has anxiety/depression issues	0.88 (0.55–1.40)	0.97 (0.65–1.44)	1.31 (0.88–1.97)	2.04 (0.81–5.15)
Currently smokes	0.73 (0.41–1.29)	0.63 (0.39–1.03)	1.08 (0.66–1.76)	0.54 (0.15–1.94)
Consumes any alcohol	1.48 (0.83–2.62)	1.40 (0.85–2.30)	**1.72 (1.03–2.88)**	1.22 (0.41–3.60)
Has a family doctor	1.26 (0.56–2.82)	1.18 (0.62–2.26)	1.33 (0.67–2.65)	2.00 (0.39–10.35)
Had fall in the past year	–	–	**2.33 (1.55–3.50)**	–

Notes: *OR* Odds ratio; *CI* Confidence Interval; bolded values indicate an odds ratio that is significant at *p*-value < 0.05; ^a^Based on objectively measured Timed Up and Go risk score

## Discussion

This study determined that in this sample of older adults (aged 55 years and older) in public housing, the prevalence of falls in the past year was 34.5%, which is substantially higher than the 19.8% observed among Canadian seniors aged 65 years and older [1]. This discrepancy in the occurrence of falls among public housing residents highlights the need for targeted interventions. One modifiable risk factor identified in this study and infrequently associated with fall history is daily consumption of fruits and vegetables, which was a protective factor in our study. Since low-income older adults are often at risk of having a poor diet, falls prevention interventions that include nutritional components or educational elements on healthy eating may be of merit. Nutritional risk has previously been identified as a determinant for falls specifically in frail older adults, [15] including factors such as ability to purchase and prepare food, unintended weight gain or loss, and other factors that could contribute to poor nutrition, however the consumption of fruits and vegetables was not part of that broad nutrition risk measure. Further research is needed to determine the extent to which diet quality can impact the postural stability of low-income older adults as well. Also, since daily consumption of fruits and vegetables was significantly associated with incidents of falls in the previous year but not with future fall risk, as measured using the TUG test, longitudinal research is needed to better understand this relationship.

Low SRHS and issues with mobility were also found to be significantly associated with history of falling in this population. These risk factors have been well-established in the literature [1, 2, 6]. Similarly, mobility issues and pain and discomfort were associated with risk of falling as objectively measured by the TUG test. Factors such as living alone, number of medications and medical conditions, including diabetes, hypertension, stroke, and depression, were identified as risk factors for falls in the literature [6] but were non-significant in this study. This is likely due to the greater influence asserted by mobility and pain and discomfort, which may have masked the association of other variables.

The outcome variable of seeking medical attention for falls has not been extensively studied however the findings followed our expectations, particularly wherein females were more likely to seek medical care for falls. This is a general trend seen for healthcare utilization across all age groups and health concerns [16, 17]. In addition, women presenting at the emergency department following a fall are more likely to have a fracture or other injury requiring hospitalization, compared to men [18]; therefore, injury severity may contribute to sex differences in medical seeking behaviour following a fall. Similarly, the association of lower SRHS with greater odds of seeking medical attention after a fall may be explained by the fact that individuals with poorer health and chronic diseases are more likely to sustain severe injury during a fall [2]. SRHS has been widely reported as an important predictor of mortality and functional decline [19, 20]. Consequently, it may be beneficial to assess older adults (55 years and older) with lower SRHS for fall risk as part of routine primary care.

Although fear of falling estimates in the literature are much more variable, ranging anywhere from 25 to 55%, they are universally reported to be more prevalent in women and with increasing age [21–23], as was found in this study. Consistent with our results, self-reported health status and a history of falls are also commonly associated with a fear of falling [21, 22, 24]. A new finding in this study was the association between alcohol consumption and fear of falling among older adults in public housing. There is very limited research available on the association between alcohol consumption and fear of falling, and none with older adults. In Canada, older adults in the general population have a lower prevalence of alcohol use (71.1%) compared to those 15–54 years of age (79.9%), but those who do consume alcohol are more likely to drink daily, compared to younger individuals (11.0% versus 4.0%) [25]. Research suggests that older adults may use alcohol for medicinal purposes [25, 26]. The prevalence of alcohol use among older adults in this public housing population was much lower (19.6%), but the strong association of their alcohol use with fear of falling suggests that this relationship warrants further research.

A strength of this study is the ability to access and evaluate a hard to reach population. This study addresses a gap in the literature by identifying risk factors for falls among older adults of low SES and uses an objective validated measure of fall risk. Another strength of the study is the wide geographical representation of older adults in public housing throughout the province of Ontario.

Limitations of this study include a small sample size of 62 participants that were identified by the TUG test as being at risk of falling. This was in part because not all participants who were positively screened through the three questions performed the TUG test (e.g. participant refusal or uses a wheelchair outside of their apartment), which may have led to an underestimation of the fall risk in this population. Moreover, cognitive impairments, which have shown to be significantly associated with fear of falling [27], were not assessed. Additionally, there have been inconsistent findings regarding the cutoff point and the ability of the TUG test to identify individuals at high risk of falls among different settings and populations [28]. However, it has been reported that the TUG test is of greater use in a less healthy and lower functioning population of older adults, similar to the participants in our study [29]. Finally, the cross-sectional design of this study prevents the temporality of associations from being established; for example, it cannot be determined whether fear of falling leads to a poor perceived health status or vice versa.

## Conclusion

Falls in older adults are a complex public health issue that require a multifaceted approach to identify the risk factors and develop effective interventions to target these populations. This study presents novel findings conducted among older adults residing in public housing, a predominantly low-income population that has largely been excluded from previous falls research. Our findings indicate a greater prevalence of falls and fear of falling exist among this population, suggesting that SES is intricately linked to an individual’s risk of falling. Moreover, the unique risk factors that may predispose individuals to falls offer insight into the multifactorial underpinnings of falls. Consequently, as the Canadian population continues to age, further research that informs interventions tailored towards the social determinants of health of low-income older adults is needed.

## Data Availability

The data that support the findings of this study are not publicly available due to them containing information that could compromise participant privacy. De-identified, limited data will be shared by the lead author upon request.

## Abbreviations

ED: Emergency department
HRQoL: Health-related quality of life
SES: Socioeconomic status
SRHS: Self-reported health status
TUG: Timed-up-and-go
